# Protocol for Nanodisc single-molecule pull-down assay to detect protein-lipid interactions

**DOI:** 10.1016/j.xpro.2026.104377

**Published:** 2026-02-17

**Authors:** Adriana Reyes-Ordoñez, Shweta Shree, Stephen G. Sligar, Jie Chen

**Affiliations:** 1 Department of Cell and Developmental Biology, University of Illinois at Urbana-Champaign, Urbana, IL, USA; 2 Department of Biochemistry, University of Illinois at Urbana-Champaign, Urbana, IL, USA; 3 Department of Chemistry, University of Illinois at Urbana-Champaign, Urbana, IL, USA; 4 Center for Biophysics and Computational Biology, University of Illinois at Urbana-Champaign, Urbana, IL, USA; 5 Cancer Center at Illinois, University of Illinois at Urbana-Champaign, Urbana, IL, USA; 6 Department of Biomedical and Translational Sciences, Carle Illinois College of Medicine, University of Illinois at Urbana-Champaign, Urbana, IL, USA

**Keywords:** Biophysics, Single-molecule Assays, Cell Biology, Cell culture

## Abstract

Phospholipids function as signaling molecules regulating numerous processes within the cellular environment. Identifying and characterizing the protein players involved in lipid-mediated functions remains a central focus. Here, we present a protocol for evaluating lipid-protein interactions using mammalian whole-cell lysates expressing proteins of interest in a single-molecule pull-down (SiMPull) assay. We describe steps for preparing materials and slide chambers, performing SiMPull, and analyzing data. Nanodiscs and total internal reflection fluorescence (TIRF) microcopy enable the detection of interactions at single-molecule resolution.

For complete details on the use and execution of this protocol, please refer to Arauz et al.^1^ and Reyes-Ordoñez et al.^2^

## Highlights

• Procedures for assembling and purifying lipid Nanodiscs
• Steps for preparing quartz slide: cleaning, passivating, and immobilizing Nanodiscs
• Instructions for performing TIRF microscopy to capture protein-Nanodisc interactions
• Guidance on single-molecule data processing and analysis

## Abstract (editor highlights)

Publisher’s note: Undertaking any experimental protocol requires adherence to local institutional guidelines for laboratory safety and ethics.

## Graphical abstract

**Figure:**
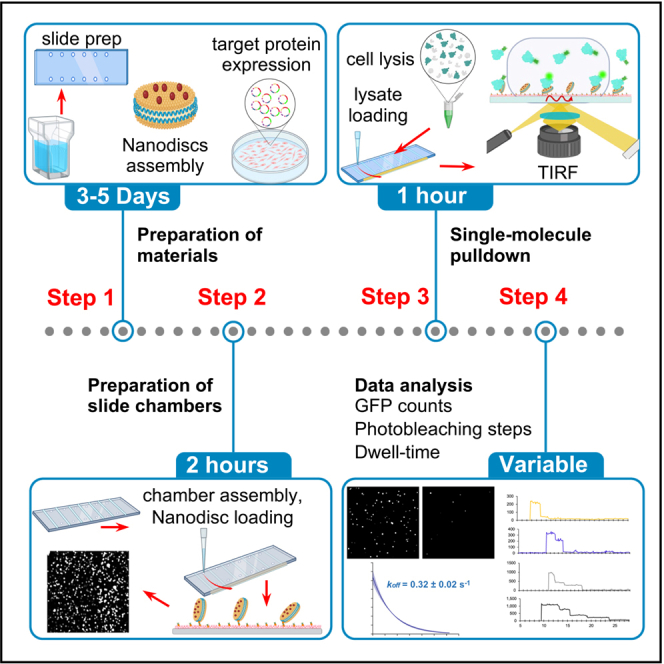

## Before you begin

### Innovation

The lipid single-molecule pulldown (lipid-SiMPull) assay was initially developed in 2016,^1^ where we used whole-cell extracts expressing fluorescently tagged proteins and small unilamellar vesicles (SUVs), and visualization of lipid-protein interaction was achieved by total internal reflection fluorescence (TIRF) microscopy. In that study known lipid-binding domains and proteins were used to demonstrate the specificity of detection. Later, we successfully applied this assay to the investigation of the family of PH-domain containing proteins for their interactions with phosphatidylinositides (or PIPs).^3^ Circumventing the need for protein purification, the assay enabled us to study full-length proteins in a near-native cellular context, which led to the striking discovery that human PH domain-containing proteins bind PIPs with a specificity much higher than previously believed in the field.^2^ However, SUVs have a severe membrane curvature, they are relatively unstable, and they present multivalent interactions that preclude single-molecule analysis. To overcome those drawbacks, recently we modified the lipid-SiMPull assay by incorporating Nanodiscs into the workflow.^2^ With this adaptation, the assay retained the specificity and sensitivity previously characterized with SUV while achieving true single-molecule resolution.

Here we provide detailed procedures of the Nanodisc SiMPull assay. This method requires significant upfront preparation, which includes (1) surface functionalization of slides for lipid immobilization, (2) assembly of Nanodiscs, and (3) expression of fluorescently labeled proteins in mammalian cells. Once these components have been prepared, the assay itself is straightforward and yields results rapidly. However, deep analysis of the data can be time consuming.

### Preparation of slides used for lipid immobilization

**Timing: 1–2 days**

The following procedure is based on a published protocol^4^ with minor adjustments.

**Figure 1:**
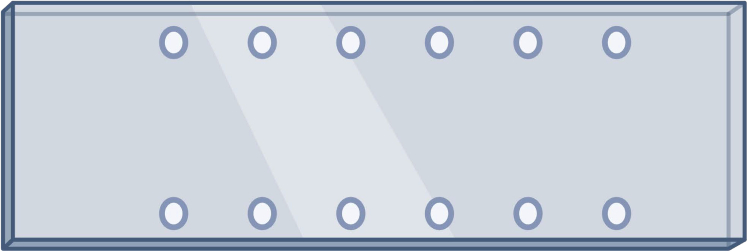
Diagram of a quartz slide with holes for sample-loading Created using Biorender.com.

***Note:*** The procedure can be paused after this step for any length of time.

1. Drill evenly separated holes along the long edges of a quartz slide. The holes should be about 0.75 mm in diameter, 3–4 mm from the edge (Figure 1, Methods video S1).

2. Clean the slides:
  a. Place the slides and coverslips in a glass Coplin jar, and bath-sonicate as follows:
      In ultrapure water for 10 min.
      In methanol for 10 min.
      In acetone for 10 min.
      In ultrapure water for 2 min.
      In methanol for 10 min.
      In ultrapure water for 1 min.
  b. Burn each slide using propane torch for at least 1 minute on each slide, until all the residues are burnt away.
  c. Return slides to Coplin jar and bath-sonicate as follows:
      In HCl:methanol (1:1) for 30 min.
      In methanol for 2 min.
    **CRITICAL:** In between each sonication step, rinse slides once in the jar using the solvent to be used in the next step (water, methanol or acetone).
3. Activate slide surface:
  a. Bath-sonicate in 2 M KOH for 30 min.
  b. Mix 100 mL of methanol with 4 mL of acetic acid, add 1.5 ml aminosilane, mix and immediately pour this solution into the jar containing slides/coverslips. Incubate in dark for 10 min at RT.
  c. Bath-sonicate for 1 minute and then return to the dark for 10 min.
4. Passivate slide surface:
  a. Prepare PEG solution: for each slide/coverslip pair, dissolve 23 mg of mPEG and 0.7 mg of biotin-PEG in 100 μL freshly prepared 10 mM sodium bicarbonate (pH 8.5). Spin in a microcentrifuge for 30 sec at 10,000*g* at 22°C–24°C to remove bubbles.
  b. Apply 100 μL of the PEG solution to one side of each slide, spread the solution evenly between the holes by tilting the slide, and immediately cover it with a cleaned coverslip.
  c. Incubate the slides in a humidified chamber, protected from light, for 3–4 hours (or up to 24 hours), at 22°C–24°C.
  d. Label the un-PEGylated side of each slide-coverslip pair, and then separate them. Rinse the entire surface of the slide thoroughly with ultrapure water (∼500 mL), and blow-dry using compressed nitrogen gas.
  e. Insert each slide/coverslip pair in a 50-mL conical tube and seal the tube using a food-grade vacuum sealer.
  f. Store at −20°C until use.

Methods video S1. Drilling of slide Preparation of slides used for lipid immobilization, related to step 1.

### Preparation of Nanodiscs

**Timing: 2 days**

The principle of Nanodisc assembly has been reported.^5^ The protocol below is for making MSP1D1-based Nanodiscs (stokes hydrodynamic diameter = 9.5nm) that contain 75 mol% 1,2-dimyristoyl-sn-glycero-3-phosphocholine (DMPC), 15 mol% 1,2-dimyristoyl-sn-glycero-3-phosphoserine (DMPS), and 10 mol% phosphoinositide (PIP).

5. Lipid mixture preparation.
  a. In a glass tube, mix the lipids dissolved in chloroform: 1.069 μmol DMPC, 0.214 μmol DMPS, 0.142 μmol PIP using Hamilton syringes.
    ***Note:*** The molar ratio of total lipids to protein is 95:1, which is higher than the typical Nanodisc lipid-to-protein ratio of ∼80:1. This excess of lipids ensures complete assembly of the Nanodiscs.
    ***Note:*** Lipids can be purchased pre-dissolved in solvent. For powdered lipids, upon reconstitution concentrations are determined by phosphate analysis. Stock phospholipid solutions (50–100 mM in solvent) are stored long-term at −20°C in glass vials (e.g., 4 mL) with PTFE-lined screw caps.
    ***Note:*** For Nanodiscs using other MSP1 variants or different types of lipids, the lipid to protein ratio and the incubation temperature for disc assembly will be different. Suggestions can be found here: https://publish.illinois.edu/sligar-lab/nanodiscs-technology-protocols-for-preparation-of-nanodiscs/.
    **CRITICAL:** Accurate calculation of lipid, MSP and buffer volumes is essential for successful disc assembly.
  b. Dry the lipids slowly under N_2_ stream while rotating the tube at an angle to form a uniform and thin film.
  c. Place tube in a vacuum desiccator for at least 4 hours, up to 24 hours at 22°C–24°C.
    **CRITICAL:** The lipid mixture must be fully dried before proceeding to the next step.
  d. Add 100 μL of reconstitution buffer (20 mM Tris-Cl, 100 mM NaCl, pH 7.4) to the dried lipids.
  e. Add sodium cholate (0.2 M) up to 40 mM final concentration.
    ***Note:*** Final cholate concentration in the reconstitution mixture should be 12–40 mM.
  f. Vortex and warm up the solution with hot tap water (∼55°C) for a few min.
  g. Sonicate in a bath sonicator briefly until solution is clear with no deposits visible on the tube wall (30 sec - 2 min).
  h. Add 0.015 μmol MSP1D1, and incubate at 22°C–24°C for 15 min.
    ***Note:*** Incubation temperature and time vary for different types of lipids. Please refer to Ritchie et al.^6^ for lipids other than those described in this protocol.
6. Nanodisc assembly.
  a. Transfer the lipid/protein mixture to a microtube containing 100-150 μL of pre-soaked Amberlite Beads XAD-2.
    **CRITICAL:** Amberlite beads must be soaked in distilled water before use.
  b. Incubate on an orbital shaker for 2 hour or up to 24 hours at 22°C–24°C.
    ***Note:*** Incubation temperature varies for different lipids as mentioned earlier.^6^
  c. Centrifuge for 1 min at 500–1,000 g to collect amberlite beads at the bottom of the tube, and transfer supernatant to a fresh tube.
  d. Pass the supernatant through a 0.2-μm bio-inert membrane to remove remaining amberlite beads.
  e. Store at 4°C until use.
7. Nanodisc purification.
  a. Size exclusion chromatography:
     Column: Superdex 200 Increase 10/300 GL.
     Buffer: PBS, pH7.4 (degassed).
     Flow rate: 0.75 mL/min.
     Fraction: 0.375 mL.
    **CRITICAL:** Buffer degassing is important for preventing bubble formation during chromatography.
  b. Collect and combine all fractions containing pure Nanodiscs (based on the protein peak).
  c. Determine Nanodisc concentration: measure A_280_; [MSP1D1] = A_280_/(23,950 x path length in cm) (M); [Nanodisc] = [MSP1D1]/2.
    ***Note:*** We typically obtain concentrations ∼7.5 μM.
8. Nanodisc storage.
  a. Short-term storage: at 4°C, 2–3 weeks.
  b. Long-term storage: add 15%–20% (v/v) of an 80% glycerol solution, aliquot the sample, flash-freeze in liquid nitrogen, and store at −80°C. Before use, thaw and repeat purification (step 7). The Nanodisc concentration will be lower.
9. DiD Labeling of Nanodiscs.
  a. Mix 1,1′-dioctadecyl-3,3,3′,3′-tetramethylindodicarbocyanine (DiD) with Nanodiscs at 10:1 molar ratio of DiD to Nanodiscs.
  b. Incubate at 37°C for 1–1.5h.
  c. Store at 4°C, use within 2–3 weeks.

### Expression of fluorescently labeled proteins (e.g., eGFP-fusion) in mammalian cells

**Timing: 2–5 days**

10. Culture HEK293 (or HEK293T) cells in high glucose DMEM supplemented with 10% fetal bovine serum, 1% Pen/Strep and 2 mM L-glutamine.
11. Seed the cells in a 6-well cell culture plate, aiming for 60%–70% confluence at time of transfection.
12. Next day, transfect the cells with plasmid expressing the eGFP-fusion protein of interest, using transfection method of your choice. We use PEI as previously reported.^7^
13. For most proteins, transfection for 18–24 hours is sufficient before lysate preparation.

## Key resources table

**Table:** 

REAGENT or RESOURCE	SOURCE	IDENTIFIER
**Chemicals, peptides, and recombinant proteins**		

Methanol (ACS grade)	Avantor Sciences	Cat# MK882006
Acetone (Certified ACS)	Fisher Scientific	Cat# A18-4
Hydrochloric acid 36%–38% (ARISTAR® ACS)	Avantor Sciences	Cat# BDH3128
Potassium hydroxide (ACS analytical reagent)	Avantor Sciences	Cat# BDH9262
Aminosilane (N-(2-Aminoethyl)-3-aminopropyltrimethoxysilane)	Millipore Sigma	Cat# 8191720100
Acetic acid glacial ≥99.7% (ACS)	Avantor Sciences	Cat# BDH3092
mPEG-SVA, MW 5,000	Laysan Bio	Cat# MPEG-SVA-5000
Biotin-PEG-SVA, MW 5,000	Laysan Bio	Cat# Biotin-PEG-SVA-5000
Sodium bicarbonate	Millipore Sigma	Cat# S6014
HEPES	Millipore Sigma	Cat# H3375
Sodium chloride	Millipore Sigma	Cat# S9888
β-Glycerophosphate disodium salt hydrate	Millipore Sigma	Cat# G9422
Sodium pyrophosphate decahydrate	Millipore Sigma	Cat# 71505
EDTA disodium salt dihydrate	Millipore Sigma	Cat# E5134
Tris base	Millipore Sigma	Cat# T1503
Protease Inhibitor Cocktail (PIC)	Roche/Sigma	Cat# 11873580001
Polyethylenimine (25 kDa, linear)	Polysciences	Cat# 23966
Amberlite XAD-2 beads	Millipore Sigma	Cat# 10301
Sodium cholate hydrate	Millipore Sigma	Cat# C6445
DMPC	Avanti Polar Lipids	Cat# 850345C
DMPS	Avanti Polar Lipids	Cat# 840018
POPC (16:0–18:1 PC)	Avanti Polar Lipids	Cat# 850457
POPS (16:0–18:1 PS)	Avanti Polar Lipids	Cat# 840034
Cholesterol	Avanti Polar Lipids	Cat# 700100
PI(4,5)P_2_ (18:1)	Avanti Polar Lipids	Cat# 850155
PI(3,4,5)P_3_ (18:1)	Avanti Polar Lipids	Cat# 850156
PI(3,4)P_2_ (18:1)	Avanti Polar Lipids	Cat# 850153
PI(3,5)P_2_ (18:1)	Avanti Polar Lipids	Cat# 850154
PI(3)P (18:1)	Avanti Polar Lipids	Cat# 850150
PI(4)P (Brain)	Avanti Polar Lipids	Cat# 840045
PI(5)P (18:1)	Avanti Polar Lipids	Cat# 850152
Biotin-PE	Avanti Polar Lipids	Cat# 870277
DiD dye	Thermo Fisher	Cat# D307
NeutrAvidin protein	Thermo Fisher Scientific	Cat# PI31000
EGFP (Enhanced Green Fluorescent Protein), recombinant	ProsPec Bio	Cat# PRO-1606

**Experimental models: Cell lines**		

HEK293T (human embryonic kidney)	ATCC	Cat# CRL-3216

**Recombinant DNA**		

pEGFP-AKT-AH	Addgene (gift from Julian Downward)	Addgene plasmid #39533
pEGFP-AKT	Addgene (gift from Julian Downward)	Addgene plasmid #39531
2PH-PLCδ-GFP	Addgene (gift from Sergio Grinstein)	Addgene plasmid #35142
pJSK659 (EGFP-SnxA)	Addgene (gift from Jason King)	Addgene plasmid #205128

**Software and algorithms**		

MATLAB (R2014b–R2024a)	MathWorks	RRID:SCR_001622
IDL 6.0	L3Harris Geospatial	
ImageJ/Fiji	NIH	RRID:SCR_002285
Custom IDL scripts (for pka file processing)	This paper/Zenodo	DOI: 4925616
Custom MATLAB scripts (distribution_CKfilt.m, Transient_dwell.m)	This paper/Zenodo	DOI: 17394218

**Other**		

Quartz microscope slides	Chemglass/Fisher Scientific	Cat# CGQ-010
Cover glasses, No. 1.5H	VWR International	Cat# 48393-241
Drill	Dremel	Cat# 3000-15
Diamon drill tip, .75mm	Crystalite Corporation	Cat# C5250510
Glass Coplin staining jar for slides	Cole-Palmer	Cat# UX-48585-20
Glass Coplin staining jar for coverslips	Fisher Scientific	Cat# ICN17006201
Beckman microtubes	Beckman Coulter	Cat# 357448
Superdex 200 10/300 GL column	Cytiva	Cat# 17-5175-01
Pall centrifugal device (0.02 μm)	Pall Corporation	Cat# ODM02C34
Ultracentrifuge with TLA 100.3 rotor	Beckman Coulter	Model TLA-100.3
Probe sonicator	Fisher Scientific	Model FB-120
Bucket sonicator (for vesicle preparation)	Laboratory Supplies Inc.	Model G112SP12
Branson Bransonic® M Mechanical Bath 2800 (for slide cleaning)	Branson Ultrasonics/Emerson	Model 2800
96-well black plate (flat bottom, non-treated) (to read gfp concentrations on cell lysates)	Corning Inc.	Cat# BTLXSF1
Agilent Synergy LX plate reader	Agilent Technologies	Cat# SLXASN
ÄKTA Pure Chromatography System	Cytiva	Model ÄKTA Pure (GE Healthcare/Cytiva)
Total Internal Reflection Fluorescence (TIRF) Microscope (Nikon Eclipse Ti-E) equipped with 488 nm and 561 nm lasers, a 100× 1.45 NA oil objective and an Andor iXon Ultra EMCCD camera	Nikon/Andor Technology/Olympus	Custom-built system

## Materials and equipment

**Table:** Sodium bicarbonate solution (10 mM)

Reagent	Final concentration	Amount
ultrapure water	N/A	10 mL
sodium bicarbonate	10 mM	8.7 mg
**Total**	**N/A**	**10 ml**

Prepare immediately before use.

**Table:** T50 buffer

Reagent	Final concentration	Amount
ultrapure water	N/A	450 mL
NaCl (1 M)	50 mM	25 ml
Tris (1 M)	10 mM	5 ml
**Total**	**N/A**	**500 ml**

Adjust pH to 8.0 and then adjust final volume to 500 ml.

Filter-sterilize using 0.22 μm filter (optional) and store at 22°C–24°C for 6–2 months (if sterilized) or 1–2 months (if not sterilized).

**Table:** Detergent-free buffer (DFB) for cell lysis

Reagent	Final concentration	Amount
ultrapure water	N/A	36.3 mL
HEPES PH 8.0 (0.5 M)	40 mM	4 mL
NaCl (1 M)	150 mM	7.5 mL
beta-glycerophosphate (0.5 M)	10 mM	1 ml
sodium pyrophosphate (0.5 M)	10 mM	1 mL
EDTA (0.5M)	2 mM	0.2 mL
**Total**	**N/A**	**50 mL**

Filter-sterilize using 0.22 μm filter and store at 4°C temperature for 1–2 months.

Just before use, add proteinase inhibitor cocktail (PIC) to the volume of buffer you intend to use. For example: add 1 μL of 100x PIC for each 100 μL of DFB to be used.

**Table:** Reconstitution buffer

Reagent	Final concentration	Amount
ultrapure water	N/A	80 mL
Tris pH 7.4 (0.5 M)	20 mM	4 mL
NaCL (1 M)	100 nM	10 mL
EDTA (0.5 M)	0.5 nM	0.1 mL
**Total**	**N/A**	**100 mL**

Filter sterilize using 0.22 μm filter and store at 4°C temperature for 3–6 months.

Bring to 22°C–24°C before use.

**Table:** Sodium cholate stock (200 mM)

Reagent	Final concentration	Amount
ultrapure water	N/A	80 mL
sodium cholate	200 mM	8.6 g
**Total**	**N/A**	**100 mL**

Stir until cholate is fully dissolved (may take 10–20 min).

Filter-sterilize using 0.22 μm filter and store at 4°C up to 3 months. Bring to 22°C–24°C before use.

**Table:** PEI solution

Reagent	Final concentration	Amount
ultrapure water	N/A	90 mL
PEI (linear)	1 mg/mL	100 mg
**Total**	**N/A**	**100 mL**

Slowly add HCl to lower the pH to 2–3, which helps dissolve PEI. Stir gently at 22°C–24°C (or at 55°C) until PEI is fully dissolved. Once fully dissolved, adjust pH to 7–7.4, and then adjust volume to 100 mL.

Filter-sterilize using a 0.22 μm filter.

Store in aliquots at −20°C (stable for >1 year). Working aliquots can be kept at 4°C for 1–2 months.

## Step-by-step method details

### Preparation of slide chambers

**Timing: 1 h**

Here we describe how to create multiple chambers on a quartz slide. These chambers will be used later for loading cell lysates in the assay.

**Figure 2:**
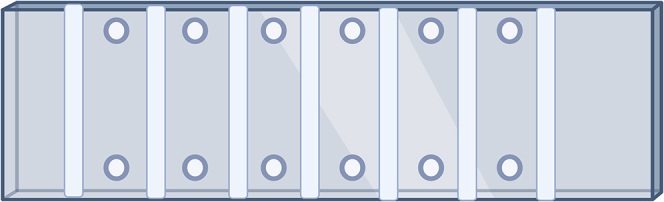
Diagram of a quartz slide taped to create chambers Created using Biorender.com.
  b. Place a coverslip on top of the taped slide, with the passivated side of coverslip facing the slide. Apply gentle pressure with a pipet tip to ensure that all tape strips are tightly adhered to both slide and coverslip.
  c. Apply a thin layer of fast-drying Epoxy along both edges of each chamber. The goal is to seal the ends of the chamber without blocking the holes for sample-loading. Allow the epoxy to fully dry before continuing.
  d. Prepare a 200 μg/mL solution of neutrAvidin protein, and flow 60 μL into each chamber to be used. Incubate 10 min at 22°C–24°C.

***Note:*** Dilute the stock of neutrAvidin using T50 buffer.

1. Constructing slide chambers.
  a. Using a sharp blade, cut narrow strips from a double-sided tape. Place the strips on the passivated side of the slide as shown in Figure 2. The width of each chamber (the space in between tape strips) should be at least 5 mm.

2. Immobilizing Nanodiscs on slide:
  a. Dilute labeled Nanodisc stock with T50 buffer, using 100-fold dilution as a starting point.
  b. Flow 80 μL of the diluted Nanodiscs into a slide chamber, incubate for 10 min, and then flow 80 μL of T50 buffer to remove unbound discs.
  c. Image on TIRF microscope in the DiD channel (638 nm), capture a couple of images (20-frame images at a 10 frame/sec rate) and determine the number immobilized Nanodiscs, aiming for 550–750 counts per field.
  d. If the disc count is too low, make a new Nanodisc dilution at a higher concentration, and repeat Step 2b in the same chamber. If the disc count is too high, dilute the Nanodisc stock further and repeat Step 2b using a new chamber.
  e. Once the DiD count is optimal, capture 10 images (20-frame images at a 10 frame/sec rate).
  f. Switch to the GFP channel (488 nm), and acquire 20-frame images at a 10 frame/sec rate in at least 10 viewing areas per chamber to achieve the desired resolution while minimizing photobleaching.^8^ These images will be processed as the background for the assay.

### Preparation of cell lysates

**Timing: 1 h**

HEK293 cells transiently expressing a GFP fusion protein of interest will be lysed by mechanical disruption in a detergent-free buffer. Concentration of the lysate will be determined.

3. Cell Lysis:
  a. Wash the transfected cells once with ice-cold PBS.
  b. Add 300 μL of DFB (with PIC freshly added) to the cells, and plate the plate on ice for 1–2 min.
  c. Transfer the cell suspension to a microcentrifuge tube. Use cell scraper to help recover all cells.
  d. Lyse the cells by passing the cell suspension through a 26-gauge needle 15–20 times.

***Note:*** Avoid excessive foaming of the samples when making the cell lysates. Lysis can also be done by a brief (3–5 s) sonication using a probe sonicator.

4. Clearing cell lysates:
  a. Ultracentrifuge the cell lysates at 90,500 g for 30 min at 4°C.
    **CRITICAL:** Only use tubes suitable for ultracentrifugation.
  b. Transfer the cleared lysate to a fresh tube, and store on ice until use.
    **CRITICAL:** Use cell lysates as soon as possible and not more than 1 h after preparation.
5. Measuring concentration of eGFP in cell lysate:
  a. Load 50 μL of PBS into each well to be used on a 96-well plate.
    ***Note:*** Use black plates optimized for fluorescence measurement.
  b. Add 50 μL of cleared cell lysate to each well and mix with the PBS.
    ***Note:*** Take care not to create bubbles when mixing the lysates with PBS.
  c. Use DFB to prepare a series of dilutions of pure recombinant eGPF (e.g., 1, 5, 15, 50, 100,350, 700 nM), and load them to the same plate (50 μL eGFP + 50 μL PBS).
  d. Measure eGFP fluorescence in a plate reader using a 488 nm filter.
  e. Create a standard curve using the pure eGFP reading, and calculate eGFP protein concentration in the lysate using.

### Protein pull-down and image acquisition

**Timing: 30–60 min**

Cell lysates will be incubated with immobilized Nanodiscs in slide chambers to allow protein-lipid interaction to occur. Fluorescence images are acquired for the purpose of quantifying and analyzing the kinetics of the interaction.

6. Using DFB, dilute cell lysate to 5 nM eGFP.

**CRITICAL:** Prepare this dilution just before loading lysates into slide chambers; do not allow diluted lysates to sit for long.

7. Load 80 μL of diluted lysate into a slide chamber with immobilized Nanodiscs. Incubate at 22°C–24°C for a desired length of time (see below).
8. Focus the microscope in the DiD channel, then switch to GFP channel and acquire a minimum of 10 images for each chamber. Capture 20-frame images at a 10 frame/sec rate.

***Note:*** Switching fields requires a quick re-focusing of the image, and some frames can be blurry. Capture more images to ensure you have at least 10 good-quality images for analysis.

9. Capture GFP images after 5, 10, 20, 30 min of incubation (longer if necessary) to determine the time needed to achieve maximal binding.

***Note:*** We found most proteins to reach maximal binding between 5 and 20 min.

10. For photobleaching step analysis or dwell-time analysis, capture ∼50 frames in the DiD channel, followed by capturing 250–350 frames in the GFP channel.

**CRITICAL:** For these types of analyses, make sure there are only 100-300 eGFP spots per field. Too many spots will interfere with the single-molecule analysis. Dilute the cell lysate below 5 nM if necessary.

### Data processing and analysis

**Timing: 30 min to many hours**

Here we describe detailed steps of analyzing the image data obtained above. GFP spot counts, photo-bleaching steps, and dissociation rate constants can be derived from the analyses.

**Figure 3:**
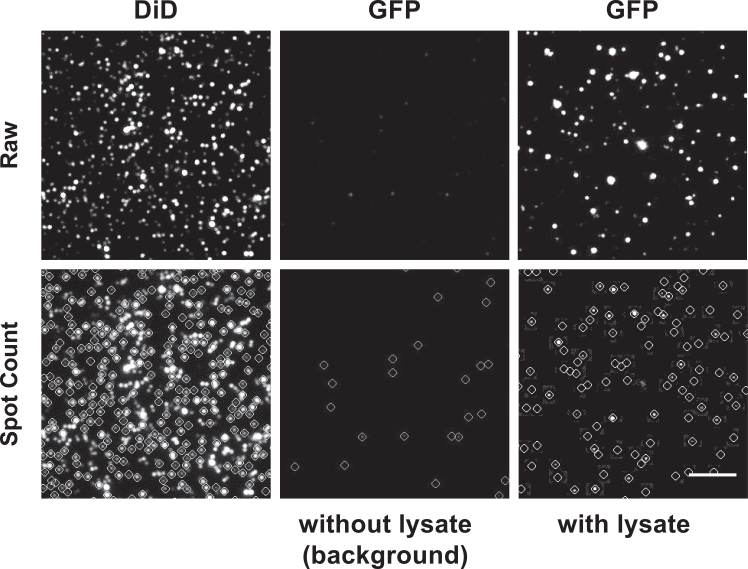
SiMPull images Examples of 20-frame TIRF images obtained before (top row) and after (bottom row) automated spot counting. DiD and GFP images (before and after lysate addition) are shown. Scale bar: 5 μm.
  b. The IDL data files above are imported into MATLAB, where each ***.pks*** file is used to calculate a spot count.
    ***Note:*** The script Count_gfp_spots.m is publicly available: https://doi.org/10.5281/zenodo.17394218.
  c. The data for each assay is the average GFP spot count difference between after and before lysate addition.
12. Determining binding threshold:
  a. Collect assay data from as many negative controls as possible (e.g., Nanodiscs without PIP + a known PIP-binding protein; Nanodiscs containing a PIP + a protein known to not bind that PIP).
    ***Note:*** We typically use data from >100 assays.
  b. Calculate the mean and standard deviation (SD) of the data above, and set the threshold of binding to mean + 2x SD. An assay data above the threshold is considered positive for binding. For instance, we have recently reported a detection threshold of 80 for our Nanodisc-SiMPull assay.
    ***Note:*** This assay yields a binary outcome (binding/no binding). Affinity of protein-Nanodisc interaction cannot be determined or inferred from the data.
    ***Note:*** The binding threshold only needs to be determined once if microscope settings and general assay conditions stay the same.
13. Photobleaching-step analysis:
  a. Process the raw images using the “ana_all_brief” script in the IDL script package for automated background subtraction and Gaussian fitting.
  b. Import the IDL output files to MATLAB, and analyze the traces containing fluorescent signals above a user-specified background (for DiD or GFP) to classify them based on their photobleaching patterns. For reference, the MATLAB scripts for the following workflow are publicly available https://doi.org/10.5281/zenodo.17394218.
    i. Use “view_traces_New”, to visualize the traces in the file and identify both the initial time point for GFP signal as well as a suitable background level which can be used to discern real DiD and GFP signals.
    ii. Using both the background values and GFP signal start points, run the “distribution_CKfilt” code to classify the trace files into files containing only GFP signal, only DiD signal and both GFP and DiD signals. Once distributed into separate folders, each trace is then processed using Chung-Kennedy filtration to smooth the signal and a png image of each trace is produced to help with classification of bleach steps.
    iii. Manually classify the bleach steps patterns observed of the initial GFP signal observed on the png files for each one of the traces located on the folder labeled as “both”.

***Note:*** As a reference for converting photobleaching steps to oligomeric states, monomeric GFP and tandem dimeric GFP (or higher order of GFP oligomers) can be pulled down using biotinylated anti-GFP antibody, followed by photobleaching step analysis. See Jain et al. for an example of this analysis.^9^

***Note:*** Be aware that blurry movies and too many particles per field tend to result in a falsely high percentage of multi-step photobleaching.

***Note:*** For automation of the process of identifying photobleaching steps, please refer to the recent report by Mattamira et al.^10^

11. Counting GFP spots:
  a. After image acquisition two files are obtained from the camera software: a ***.log*** file, detailing the time and conditions used for the file acquisition and a ***.pma*** file containing the raw images and all metadata associated with the file. Process these raw images using the IDL software and the script package publicly available (https://doi.org/10.5281/zenodo.4925616).
    ***Note:*** This script uses point-spread function (PSF) fitting to localize individual fluorescent molecules in the image and outputs files containing the coordinates and intensities of all detected spots: the ***.pks*** file contains a particle list with their respective coordinates; the ***.trace*** file contains fluorescence intensity vs time for each spot; two ***.tif*** image files contain the average projection of all the frames in the movie with and without the detected spots marked (with circles) (Figure 3) to help visualize which molecules were chosen for trace extraction. For automated analysis of multiple files, the “ana_all.pro” script runs through the entire workflow automatically.
    ***Note:*** The file format described here is specific to the Andor-based acquisition workflow used on our microscope. For any other type of workflow, ensure that image stacks are saved in the original format to preserve time-resolved information for kinetic analysis.

14. Dwell-time analysis:
  a. The same data processed above can be used for this analysis.
  b. In MATLAB, select the traces that display transient binding events (i.e., re-binding occurring on the same spot) and use “Transient_dwell” to manually measure their dwell-time.
    ***Note:*** Fluorescent signals immediately after 488 nm laser excitation should not be included in dwell-time measurement (see Figure 6A).
  c. Dwell-time data are used to calculate the dissociation rate constant (*k*_*off*_) using an exponential decay model (i.e., maximum likelihood estimation or MLE). SEM and 95% confidence intervals can be determined. A script to perform the analysis on MATLAB is available: https://doi.org/10.5281/zenodo.17394218.

## Expected outcomes

**Figure 4:**
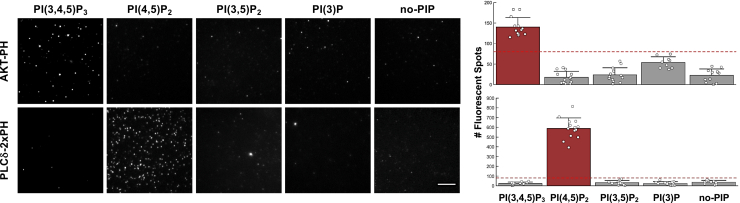
Results of Nanodisc-SiMPull assay eGFP-fusion proteins were transiently expressed in HEK293 cells, and cell lysates (5 nM eGFP-fusion) were subjected to SiMPull assay using Nanodiscs containing 5% PI(3)P, PI(4,5)P_2_, PI(3,5)P_2_, or PI(3,4,5)P_3_, or 0% PIP as negative control (no-PIP). Representative TIRF images are shown. Scale bar: 5 μm. The graphs show data of one experiment: average number of eGFP spots per image area after subtracting background counts with error bars representing standard error. The dotted lines indicate threshold for interaction (80). Figure adapted with permission from Reyes-Ordoñez et al., 2025.^2^

This assay detects protein-lipid interactions with high specificity and has a sensitivity of capturing interactions up to K_d_ of 10-20 μM.^2^ As examples, PH domains of AKT (AKT-PH) and PLCδ (PLCδ-2xPH) interacting with PI(3,4,5)P_3_ and PI(4,5)P_2_, respectively, are detected by this assay (Figure 4).

**Figure 5:**
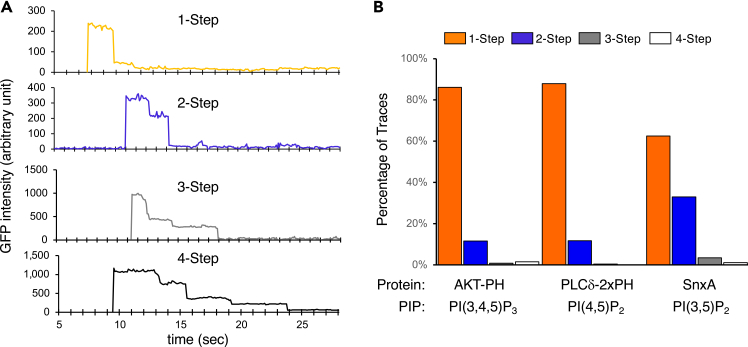
Photobleaching analysis of SiMPull assay eGFP-fusion proteins were transiently expressed in HEK293 cells, and cell lysates (5 nM eGFP-fusion) were subjected to SiMPull assays using Nanodiscs containing 5% PI(4,5)P_2_, 5% PI(3,5)P_2_, or 10% PI(3,4,5)P_3_. (A) Examples of single eGFP traces representing 1-, 2-, 3-, and 4-photobleaching steps are shown. (B) Percentage of eGFP spots that displayed each of the four photobleaching steps is shown as mean ± SEM (n = 3–7 independent assays). Figure adapted with permission from Reyes-Ordoñez et al., 2025.^2^

We have previously reported^2^ photobleaching step analysis of several protein-lipid pairs using this assay: representative traces of 1- to 4-step bleaching are shown in Figure 5A, and Figure 5B shows the percentage of each step for three protein-lipid pairs. The data indicate that AKT-PH and PLCδ-2xPH interact with their respective PIP mostly at one protein per Nanodisc, whereas the increased percentage of 2-step photobleaching for SnxA on PI(3,5)P_2_ Nanodisc is consistent with this protein forming a dimer.^11^

**Figure 6:**
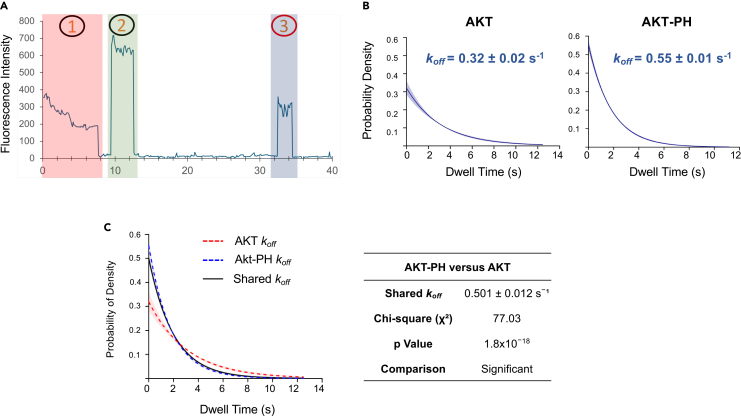
Dwell-time analysis of SiMPull assay (A) A representative single-particle fluorescence trace. 1 = DiD fluorescence. 2 = initial GFP fluorescence representing a molecule that was already bound to the Nanodisc at the moment of GFP excitation. 3 = a second protein molecule bound to the Nanodiscs with a dwell-time of 2.5 seconds. (B) Dissociation rate constants (*k*_*off*_) derived from exponential decay fitting of dwell time data for the full-length protein and the PH domain of AKT. Shaded areas represent the 95% confidence intervals. (C) Statistical comparison of the dissociation rate constants obtained for AKT and the PH domain of AKT via LTR analysis. Figure adapted with permission from Reyes-Ordoñez et al., 2025.^2^

A representative trace with transient re-binding events is shown in Figure 6A, which can be used to measure dwell-time and calculate dissociation rate constant. The dissociation rate constants for full-length AKT and AKT-PH binding to Nanodiscs containing PI(3,4,5)P_3_ are determined through dwell-time analysis (Figure 6B).

### Statistical analysis

GFP counts from this assay are only used to determine a binary outcome – binding or no binding – by comparing to the pre-determined threshold. Data from replicate experiments are not averaged and nor analyzed for statistical comparison. Nevertheless, multiple experiments should be performed with independent cell lysates expressing the protein of interest to bolster a conclusion of specific protein-lipid interaction. The dissociation rate constants obtained from fitting dwell-time data to exponential decay model can be compared between different experiments or different samples by using likelihood ratio test (LRT) (Figure 6C), to assess statistical significance of the differences found.^2^

## Limitations

While the single-molecule resolution of this assay is a significant advantage, it comes with a major limitation inherent to all single-molecule studies – the need to work with very low protein concentrations. As such, it is currently not feasible to determine dissociate constants of interactions, and the assay is not truly quantitative. Technical innovation in microscopic hardware will be necessary to overcome this limitation. Another limitation of this assay is its reliance on fluorescence-labeled recombinant proteins, precluding direct interrogation of native, endogenous proteins. The advent of gene editing tools has made it feasible to tag a protein of interest in the endogenous locus with relative ease, although knocking in a fluorescent tag and validating functional integrity still entail significant efforts.

## Troubleshooting

### Problem 1

High background GFP signals (related to step 2f).

Suboptimal preparation of the quartz slides can lead to high GFP signals before the addition of cell lysates containing GFP protein, in which case the assay should not proceed.

### Potential solutions

Repeat slide preparation paying particular attention to the following:

• Clean slides thoroughly; and do 2 cycles of slide burning.
• Make sure the aminosilane solution is not too old.

### Problem 2

It is not possible to obtain any well-focused images (related to steps 2e and 2f).

This is likely due to misalignment in the microscope, which would make performing the assay impossible.

### Potential solution

The only solution is to re-align the lasers in the TIRF microscope. Proper tools and expertise are necessary to perform the alignment, which is beyond the scope of this protocol.

### Problem 3

Positive control assay does not work.

It is important that *every* assay is accompanied by positive binding controls. In addition to the possibility of loss of Nanodisc integrity, expression and proper folding of the recombinant protein can vary from experiment to experiment, depending on cell culture status and transfection efficiency. We suggest keeping the following aspects in mind when a positive control assay does not work.

### Potential solutions

• Use a new batch of Nanodiscs (freshly assembled or newly thawed) and repeat the assay. We recommend using Nanodiscs stored at 4°C for no longer than 2–3 weeks.
• Ensure that the passage number of the cells is not too high, and that cells look healthy.
• Aim for an optimal expression level of the recombinant protein by adjusting transfection conditions. Insufficient expression and excessive overexpression can both reduce the quality of the recombinant protein in cytosol.

### Problem 4

Negative binding control is positive, or proteins bind Nanodiscs regardless of disc content.

For each experiment assaying a protein of interest, a negative control using Nanodiscs containing no target lipid (e.g., a specific PIP) should be included. Sometimes a protein may be found to bind all Nanodiscs with no specificity. Before concluding that this protein has a non-specific affinity for lipids, the possibility of recombinant protein mis-folding should be ruled out.

### Potential solutions

• Some proteins when overexpressed may not fold properly, leading to non-specific “sticking” to lipids. If the GFP fusion protein is at a very high level in the cell lysates, try transfecting less DNA and/or for a shorter time.
• The misfolding problem could also occur when cells are unhealthy. Hence, low-passage cultures and optimal growth conditions can potentially help to mitigate the problem.

### Problem 5

Inconsistent assay results for a protein of interest from experiment to experiment.

Some proteins behave very consistently in the Nanodisc-SiMPull assay (such as AKT-PH binding to PIP_3_ and PLCδ-PH binding to PI4,5P_2_), whereas others do not. Several factors can potentially contribute to the inconsistency of assay results for a protein of interest: the protein may be prone to misfolding and hence is more dependent on optimal cell culture conditions; the protein may be predominantly associated with cellular membranes and at a very low level in the cytosol; the protein-lipid affinity is near the threshold of detection for this assay (K_d_ of 10–20 μM).

### Potential solutions

• Ensure optimal health of cell culture.
• Increase the level of recombinant protein expression by adjusting transfection conditions.
• Increase the concentration of cell lysates in the assay and add a “chase” step to remove unbound GFP proteins before imaging. For instance, 50 nM (instead of 5 nM) of GFP lysates can be added to the slide chamber with Nanodiscs; after incubation for a desired period of time to allow binding, inject 80 μL of T50 buffer into the chamber and image immediately.
• It should be emphasized that this assay has a binary outcome (binding/no binding). For a protein-lipid interaction with affinity near the threshold of detection, inconsistent outcome is expected. Such an outcome is still meaningful, as it would support the conclusion that the interaction may occur at a low affinity.

## Resource availability

### Lead contact

Further information and request for resources and reagents should be directed to the lead contact, Jie Chen (jiechen@illinois.edu).

### Technical contact

Technical questions on executing this protocol should be directed to and will be answered by the technical contact, Adriana Reyes-Ordoñez (ariyis@illinois.edu).

### Materials availability

This study did not generate new unique reagents or materials.

### Data and code availability

All data are available from the lead contact upon request.

IDL and MATLAB scripts used in this study are publicly available at GitHub and Zenodo:

• smFRET package used for SiMPull image acquisition: https://github.com/Ha-SingleMoleculeLab.
• IDL scripts used to process raw image files: https://doi.org/10.5281/zenodo.4925617.
• MATLAB scripts for GFP spot counting, bleach-step, and dwell time measurement analyses: https://zenodo.org/records/17394218.
• MATLAB scripts for maximum likelihood estimation and LRT analyses: https://doi.org/10.5281/zenodo.15313208.

## Acknowledgments

This work was supported by the National Institutes of Health (grant nos. GM089771 to J.C. and GM118145 to S.G.S.).

## Author contributions

A.R.-O.: data curation, formal analysis, methodology, data analysis/code editing and validation, and writing of the original draft of the manuscript and editing; S.S.: Nanodisc assembly methodology and editing of the manuscript; S.G.S.: conceptualization, funding acquisition, and editing of the manuscript; J.C.: conceptualization, funding acquisition, and editing of the manuscript.

## Declaration of interests

The authors declare no competing interests.
